# Institutional delivery and postnatal care services utilizations in Abuna Gindeberet District, West Shewa, Oromiya Region, Central Ethiopia: A Community-based cross sectional study

**DOI:** 10.1186/s12884-016-0940-x

**Published:** 2016-07-07

**Authors:** Birhanu Darega, Nagasa Dida, Fikru Tafese, Shimeles Ololo

**Affiliations:** Department of Nursing, Goba Referral Hospital, Madda Walabu University, Bale-Goba, Ethiopia; Department of Public Health, Goba Referral Hospital, Madda Walabu University, Bale-Goba, Ethiopia; Department of Health Service Management, College of Public Health and Medical Sciences, Jimma University, Jimma, Ethiopia

**Keywords:** Institutional delivery, Postnatal care, Associated factors, Abuna Gindeberet district

## Abstract

**Background:**

Delivery at health institutions under the care of trained health-care providers and utilization of postnatal cares services plays vital roles in promoting child survival and reducing the risk of maternal mortality. More than 80 % of maternal deaths can be prevented if pregnant women access to essential maternity cares like antenatal care, institutional delivery and postnatal care services. Thus, this study aimed to assess institutional delivery and postnatal care services utilizations in Abuna Gindeberet District, West Shewa, Oromiya Regional State, Ethiopia.

**Methods:**

A community-based cross-sectional study design was employed among 703 randomly identified mothers of Abuna Gindeberet district in March, 2013. Data were collected through interviewer-administered questionnaires and analyzed using SPSS version 16.0. Descriptive, bivariate and multivariate analyses were used to determine prevalence and to identify associated factors with institutional delivery and postnatal care, considering *p*-value of less than 0.05 as significant. The results were presented in a narrative forms, tables and graphs.

**Results:**

One hundred one (14.4 %) of mothers gave birth to their last baby in health institutions. From 556 (79.1 %) of respondents who heard about postnatal care services, only 223 (31.7 %) of them utilized postnatal care services for their recent childbirth. From the total postnatal care users, 204 (91.5 %) of them took the services from health extension workers. Decision-making styles, household distances from health institutions, household being model family and ANC services utilizations were found to be statistically significant with both institutional delivery and postnatal care services utilizations. But educational status of husbands was statistically significant with only postnatal care services utilizations.

**Conclusions:**

Both institutional delivery and postnatal care services utilizations from health institutions were low. Decision-making styles, household distances from health institutions, household being model family and ANC services utilizations were the common factors that affect institutional delivery and postnatal care services utilizations from health institutions. Therefore, giving attention to the identified factors could improve and sustain institutional delivery and postnatal care services utilizations from health institutions.

## Background

Maternal mortality remains a major challenge to health systems worldwide. Some eight million women suffer from pregnancy-related complications and over half a million die annually. In developing countries, one woman in 11 may die of pregnancy related complications compared to one in 5000 in developed countries [1].

In the sub-Saharan Africa, over 162,000 women still die each year during pregnancy and childbirth. Most of these deaths are because of the lack of access to skilled childbirth attendances and emergency cares [2].

Maternal mortality in Ethiopia is high relative to developed countries and some developing countries. Every year, 500,000 maternal disabilities occur in the country [3]. Maternal mortality ratio (MMR) in 2011 was 676 deaths per 100,000 live births [4].

More than 80 % of maternal deaths can be prevented if pregnant women access essential maternity cares like antenatal care services, institutional delivery that by skilled attendance at childbirth as well as emergency obstetric care and postnatal care services [5]. Institutional delivery services utilizations in Oromiya region was 4.3 % in EDHS 2005 and increased to 8 % in EDHS 2011, which was much lower than the national level. In addition to this, the postnatal care services utilizations was also very low that is 4.8 % in EDHS 2005 and increased to 6.1 % in EDHS 2011 [4, 6]. Hence, this study conducted to determine the prevalence of institutional delivery and postnatal care service utilization and their associated factors among Abuna Gindeberet District mothers, Oromiya Regional State, West Shewa Zone, Ethiopia.

## Methods

### Study setting and participation

A community-based cross-sectional study was conducted among 703 women who gave birth in the last 12 months in Abuna Gindeberet district, West Shewa Zone, Oromiya Regional State in March 2013. The district had 42 rural sub-districts from which 14 of them randomly selected. The rationale behind selecting 14 sub-districts over 42 is to include representative sample which mean in some literature of world health organization (WHO), the sample must be between 30 and 50 % of total population. Therefore, we tried to include one-third of the total population [7]. In each selected sub-districts, households with under 1-year children identified through census and sampling frame was developed. Finally, study subjects were addressed through simple random sampling by using sampling frame developed from conducted census data [8]. The sample was determined using single population proportion formula with an assumption of level of confidence of the study 95 %, sampling error tolerated 5 % and proportion of institutional delivery care services utilization (P) 50 % used. Design effect of 1.7 and a non-response rate of 10 % were also considered. Design effect 1.5 to 2 is enough in cross sectional study which done in multistage sampling. Depending on the population under study whether the community is homogeneous or heterogeneous we can use starting from 1.5 to 2 design effect. If we consider the community is heterogeneous, we can use design effect 2. Again, if we consider the community is homogeneous, we can use design effect 1.5 [9, 10]. According to this, in this study the rural dispersed population is our study. Even though, they are dispersed population, all of them are rural. Two things considered in this condition, being all of them rural makes them homogeneous population and being dispersed population makes them heterogeneous population. Therefore, since these two conditions were present, we used design effect averagely or in middle of the range (because we can use starting from 1.5 up to 2 depending on condition of the population).

### Operational definitions

**Postnatal care**: the services given to a mother for a period of 6 weeks from the time of delivery in their previous delivery from health institutions.

**Decision making style**: It is the ways of determining and control over resources when women should seek health care services. The style may be both husband and wife decides together or separately by individual.

**Model family**: is a family that applied all health extension packages at their home and got certificates of appreciations from health extension workers.

### Instruments and data collection methods

Structured questionnaires, which address the objectives of the study, were adapted from pertinent literatures [11–13, 15–17, 18–20]. The questionnaires translated into the local language - Afan Oromo and retranslated to English. Pre-test was done on 5 % of the sample size in sub districts (kebeles) different from those selected sub-districts for the study before actual data collections. House to house, data collection was made through interviewer-administered questionnaires.

### Data processing and analysis

Data entered into Epidata version 3.1 and exported to SPSS version 16.0 for analysis. Descriptive analysis was done to determine the prevalence of institutional delivery care services and postnatal care services utilizations. Binary and multiple logistic regression analyses by backward conditional method were used to identify associated factors with institutional delivery care services and postnatal care services utilizations. Variables that had *p*-value of less than 0.05 by binary logistic regression were included in the multiple logistic regression analysis. A *p*-value of 0.05 was used as cutoff point to identify statistically significant variables.

## Results

### Socio-demographic characteristics of respondents

A total of 703 mothers were participated in the study with a response rate of 98.7 %. The mean age of the respondents was 31.5 (SD ± 7.34) years. Protestant Christianity account for the highest proportion in religion 525 (74.7 %) followed by Orthodox (17.4 %). Three hundred sixteen (45 %) of the respondents had not attended any form of educations. Six hundred eighty one (96.9 %) of the respondents were married (Table 1).

**Table 1:** Socio-demographic characteristics of the respondents in Abuna Gindeberet district, West Shewa Zone, Oromiya Region, Central Ethiopia, March, 2013

Variables	Alternatives	Number (*n* = 703)	Percent (%)
Educational status	1. No education	316	45.0
	2. Only read and write	110	15.6
	3. Primary education	233	33.1
	4. Secondary education	44	6.3
Educational level of husbands of respondents	1. Unable to read and write	174	25.6
	2. Able to read and write	507	74.4
Religion	1. Protestant	525	74.7
	2. Orthodox	122	17.3
	3. Wakefata	56	8.0
Occupation of respondents	1. Housewife	648	92.2
	2. Employed	55	7.8
Occupation of husband of respondents	1. Farmer	547	80.3
	2. Employed	89	13.1
	3. Merchant	45	6.6
Marital Status	1. Married	681	96.9
	2. Single	22	3.1
Distance of house from health post	1. 1–5 km	461	65.6
	2. >5 km	242	34.4
Household status towards as model family	1. Did not hear about model family	22	3.1
	2. Have heard but not at all working towards graduation	66	9.4
	3. Working towards graduation	472	67.1
	4. Graduated as model family	143	20.3

### Institutional delivery services utilizations

This study revealed that 101 (14.4 %) women were delivered their recent babies in health institutions and majority of women 602 (85.6 %) were delivered at home. Two hundred fourteen (35.5 %) of home deliveries were attended by mother-in-law, by their mother 138 (22.9 %) and relatives/neighbors 109 (18.1 %). Only 51 (8.5 %) of home delivery were assisted by health extension workers. The major reasons for preference of home delivery were history of normal previous home delivery 261 (43.4 %) and wanting to stay at home with their relatives 169 (28 %).

Among 101 (14.4 %) mothers that delivered at health institutions, 44 (43.6 %) of them reported that they had bad outcome with previous home delivery, 29 (28.7 %) had difficulty in labor and 23 (22.8 %) need better service were their major reasons for why they preferred delivery at health institutions. From the total mothers interviewed in the study, 498 (70.8 %) of them had an intention to deliver their newborns at health institutions in the future (Table 2).

**Table 2:** Delivery Services Utilizations in Abuna Gindeberet district, West Shewa Zone, Oromiya Region, Central Ethiopia, March 2013

Variables	Alternatives	Number (*n* = 703)	Percent (%)
Place of delivery	1. Home	602	85.6
	2. Health center and Hospital	34	4.9
	3. Health post	67	9.5
Person assisted for home delivery	1. Mother-in-law	214	35.5
	2. Relatives/neighbors	109	18.1
	3. Mother	138	22.9
	4. Health extension workers	51	8.5
	5. Health professionals	7	1.2
	6. Traditional birth attendant	83	13.8
Reason for home delivery	1. Facility not opens regularly	52	8.7
	2. Poor quality service of HFs	76	12.6
	3. Need to be with relatives	169	28
	4. Previous home deliver was normal	261	43.4
	5. I was told my pregnancy is normal	44	7.3
Reason for delivering at health facility	1. Need better service	23	22.8
	2. Bad outcome with previous home deliver.	44	43.6
	3. Difficulty during labor.	29	28.7
	4. Others	5	4.9
Decision maker at home	1. The mother herself	79	11.3
	2. Her husband	61	8.7
	3. Both of them	563	80.0
Place of delivery for the next newborn	1. At home	193	27.5
	2. Traditional birth attendant	12	1.7
	3. Health institutions	498	70.8

### Associated factors of institutional delivery service utilizations

According to this study women who decide by themselves were 2.8 times more likely to utilize delivery care from health institutions [AOR = 2.859, 95 % CI = 1.56, 5.22]. Similarly, women that live at the distance of less than 5 km away from health facility were 9.2 more likely to utilize delivery care from health institutions than those women who live at a distance of greater than 5 km away from health facility [AOR = 9.179, 95 % CI = 3.825, 22.026]. In addition; those respondents who were from model family household were 6.7 times more likely to utilize institutional delivery than respondents who were from non-model family household [AOR = 6.744, 95 % CI = 4.052, 11.226]. Those women who use ANC services were also 3.7 times more likely to use institutional delivery services than women who did not use ANC services [AOR = 3.671, 95 % CI = 1.098, 12.275].

Age, educational level of respondent and their husband, occupation of respondent and their husband were not statistically associated with institutional delivery services utilizations from health institutions in this study (Table 3).

**Table 3:** Institutional delivery services utilization and associated factors from health institutions in Abuna Gindeberet District, West Shewa Zone, Oromiya Region, Central Ethiopia, March, 2013

Socio demographic	Alternatives	Institutional delivery service utilizations		COR [95 % C.I]	AOR [95 % C.I]
		Yes	No		
Age of respondents	18–24 years	17	117	0.750 (0.0.410–1.373)	
	25–34 years	41	263	0.805 (0.506–1.280)	
	35 and above	43	222	1.00	
Educational level of respondents	Unable to read and write	38	278	1.00	
	Able to read and write	63	324	1.423 (0.922–2.194)	
Decision making styles	Wife	33	46	5.496 (3.279–9.212)	2.859 (1.565–5.223)*
	Husband	3	58	0.396 (0.396–1.301)	0.467 (0.133–1.639)
	Both together	65	498	1.00	1.00
Educational level of husbands of respondents	Unable to read and write	28	146	1.00	
	Able to read and write	73	434	2.142 (1.358–3.379)	
Model family	Not model	39	521	1.00	1.00
	Model family	62	81	10.225 (6.430–16.262)	6.744 (4.052–11.226)*
Occupation of respondent	Housewife	87	561	1.00	
	Employed	14	41	1.022 (1.556–2.879)	
Occupation of husbands of respondents	Farmer	79	468	1.00	
	Employed	13	76	1.022 (0.544–1.920)	
	Merchant	9	36	2. 013 (1.736–3.179)	
Household distance from health institutions	1–5 km	97	364	9.721 (4.191–22.546)	9.179 (3.825–22.026)*
	>5 km	7	235	1.00	1.00
Have you used ANC	Yes	98	481	8.218 (2.561–26.370)	3.671 (1.098–12.275)*
	No	3	121	1.00	1.00

*p < 0.001

### Postnatal care services utilizations

Of the total respondents, 556 (79.1 %) of them heard about postnatal care services. Two hundred twenty three (31.7 %) respondents utilized the postnatal care services for their recent child and from this, 204 (91.5 %) of them utilized the services from health extension workers. Two hundred twelve (30.2 %) of the respondents who utilized postnatal care for their recent child were from mothers who gave birth at their home. Three hundred (42.7 %) respondents said that if the problem resulted after delivery on the baby, they will go to health institutions (Table 4).

**Table 4:** Postnatal care Services Utilizations in Abuna Gindeberet district, West Shewa Zone, Oromiya Region, Central Ethiopia, March 2013

Variables	Alternatives	Number (*n* = 703)	Percent (%)
Ever heard of postnatal care	1. Yes	556	79.1
	2. No	147	20.9
Used PNC in the recent delivery	1. Yes	223	31.7
	2. No	480	68.3
Place of PNC used	1. Health post	204	91.5
	2. Other health institutions	13	5.9
	3. Traditional birth attendant	6	2.6
Visited by health worker immediately after delivery of your recent child	1. Yes	212	30.2
	2. No	491	69.8
The health workers visited you during recent delivery	1. Health extension worker	167	78.6
	2. Traditional birth attendant	41	19.3
	3. Health professionals	4	2.1
Health facilities you visit if any problems after birth	1. Stay home	23	3.3
	2. Traditional healers	78	11.1
	3. Health post	300	42.7
	4. Other health institutions	235	33.4
	5. Religious place	67	9.5

### Associated factors of postnatal care service utilizations

In this study women who used ANC services were 4.9 times more likely to use postnatal care services than women who did not use ANC services for their recent child (AOR = 4.956, 95 % CI = 2.506, 9.80). Women who able decide by themselves were 2.3 times more likely to utilize postnatal care from health institutions [AOR = 2.319, 95 % CI = 1.825, 6.520]. Similarly, women that live at the distance of less than 5 km away from health facility were 2.3 times more likely to utilize postnatal care from health institutions than those respondents who live at greater than 5 km away from health facility [AOR = 2.320, 95 % CI = 1.557, 3.455]. In addition; respondents who were from household that become model family were found to be 3 times more likely to utilize postnatal care services than respondents from households that were not model family [AOR = 2.970, 95 % CI = 1.985, 4.444].

Age, educational level of respondent, occupation of respondent and their husband were not associated with postnatal care services utilization from health institutions in this study (Table 5).

**Table 5:** Association of factors with postnatal care services utilizations in Abuna Gindeberet District, West Shewa Zone, Oromiya Region, Central Ethiopia, in March, 2013

Socio demographic	Alternatives	Postnatal care services utilizations		COR [95 % C.I]	AOR [95 % C.I]
		Yes	No		
Age of respondent	18–24 years	38	96	1.235 (0.784–1.946)	
	25–34 years	98	206	1.027 (0.723–1.460)	
	35 and above	87	178	1.00	
Educational level of respondent	Unable to read and write	98	218	1.00	
	Able to read and write	125	262	0.942 (0.684–1.297)	
Decision making style	Wife	32	47	6.241 (2.402–16.217)	2.319 (1.825–6.520)**
	Husband	6	55	1.391 (0.859–2.254)	0.835 (0.486–1.436)
	Both together	185	378	1.00	1.00
Educational level of husbands of respondents	Unable to read and write	29	145	1.00	
	Able to read and write	194	313	2.950 (1.824–4.771)	
Model family	Not model	139	421	1.00	
	Model family	84	59	4.312 (2.937–6.332)	
Occupation of respondents	Housewife	206	442	1.281 (0 0.797–2.058)	
	Employed	17	38	1.00	
Occupation of husbands of respondents	Farmer	172	375	1.00	
	Employed	37	52	1.637 (1.038–2.582)	
	Merchant	14	31	3.492 (1.681–4.078)	
Household distance from health institutions	1–5 km	172	289	2.229 (1.552–3.201)	2.320 (1.557–3.455)**
	>5 km	51	191	1.00	1.00
Have you used ANC	Yes	210	369	6.634 (3.401–12.941)	4.956 (2.506–9.80)**
	No	13	111	1.00	1.00
Institutional delivery for recent baby	yes	60	41	3.941 (2.549–6. 095)	1.853 (1.101–3.120) **
	No	163	439	1.00	1.00

**P < 0.003

## Discussion

This article has tried to assess institutional delivery and postnatal care services utilization and their associated factors among mothers of under 1 year children. From the total study participants, only 101 (14.4 %) of them delivered their youngest child in health institutions. Among the home delivery, 214 (35.5 %) attended by mother-in-law, 138 (22.9 %) attended by their mother, 109 (18.1 %) attended by relatives/neighbors and 51 (8.5 %) assisted by HEWs.

In case of the study done in Tigray region, Ethiopia; 691 (95.3 %) of women delivered their baby at home with the help of relative/friend/neighbor 586 (80.8 %) and 49 (6.8 %) were assisted by the HEWs [11]. In addition, the study conducted in Sekela District, North West of Ethiopia; indicated that 326 (87.9 %) of the mothers delivered at home with the assistance of family members 274 (80 %), themselves 20 (6.1 %), their mother 13 (3.98 %) and health workers 2 (0.6 %) [12].

Moreover, the study done in in Munisa district, South East Ethiopia show that, 750 (87.7 %) of the mothers gave birth to their last baby at home by the assistance of family or relatives 392 (52.2 %), untrained traditional birth attendant 288 (38.4 %), themselves 23 (3.1 %) and no one were assisted by health workers during their home delivery [13].

The delivery services utilizations from health institutions can be affected by distance of household from health institution, household as model family, decision-making style in household and utilization of ANC services.

In contrary to this finding, in study done at Wukro and Butajera districts, Ethiopia; the factors that determine use of institutional delivery were women education and household economic status. However, women’s autonomy in decision making on place of delivery did not show statistical association [14]. Moreover, according to the study conducted in Dodota district, Oromiya regional state, Ethiopia; residence, educational level of mothers, pregnancy related health problems, previous history of prolonged labor, and decision making style showed association with utilization of institutional delivery services utilizations [15].

Concerning the postnatal care services 223 (31.7 %) of the respondents utilized the services for their recent child. From those had utilized postnatal care services for their last child, 204 (91.5 %) of them took the services from health extension workers. This result is congruent with study done in Sidama zone, Southern Ethiopia; where 37.2 % of the mothers utilized postnatal care services from health institutions [16]. To the contrary, in Tigray region, Ethiopia; only 5 % of the mothers had PNC checkups for their baby at health institutions [11].

Postnatal care services utilization from health institution can be affected by educational status of their husband, household distance from health institution, household as model family, decision making style, ANC services utilization and place of recent delivery. In similar educational status, women’s autonomy, number of pregnancy and place of delivery were the factors making a difference in utilizing post natal care service [16]. Confidence and knowledge from previous pregnancies and births were the major reasons for low utilization of postnatal care services among many child holder women.

In this study interviewer administered method of data collection used, that has contribution for quality of data collected. The data were collected from representative sample and it can be generalized for the total population. Only quantitative methods of data collection were used and it is better if qualitative methods were included to triangulate the information collected from the sample.

## Conclusions

Both institutional delivery and postnatal care services utilizations from health institutions were low. Decision-making style, household distance from health institutions, household being model family and ANC services utilization were found to be statistically significant with both institutional delivery and postnatal cares service utilizations. Educational status of respondents’ husband is found to be statistically significant with only postnatal care services utilization from health institutions.

Thus, focusing these identified factors for individual variables could improve and sustain institutional delivery and postnatal care services utilizations from health institutions simultaneously.

## Abbreviations

ANC, antenatal care; AOR, adjusted odd ratio; COR, crude odd ratio; CI, confidence interval; EDHS, Ethiopian Demographic and Health Survey; HEWs, health extension workers; HF, health facilities; PNC, postnatal care.
